# Compliance of WHO and UNICEF estimates of national immunization coverage (WUENIC) with Guidelines for Accurate and Transparent Health Estimates Reporting (GATHER) criteria

**DOI:** 10.12688/gatesopenres.13258.1

**Published:** 2021-05-10

**Authors:** M. Carolina Danovaro-Holliday, Marta Gacic-Dobo, Mamadou S. Diallo, Padraic Murphy, David W. Brown

**Affiliations:** 1 World Health Organization, Geneva, Switzerland; 2 United Nations Children's Fund, New York, USA; 3 BCGI LLC / pivot-23.5°, Cornelius, NC, USA

**Keywords:** global health, vaccination, health status indicators, checklist, epidemiologic methods

## Abstract

**Background**: The objective of the study was to assess compliance of the WHO and UNICEF estimates of national immunization coverage (WUENIC) against the 18 criteria of the Guidelines for Accurate and Transparent Health Estimates Reporting (GATHER) that define and promote good practice in reporting of global health estimates.

**Methods**: We conducted a desk review of the WUENIC estimation and reporting process vis-à-vis each of the 18 GATHER criteria to complete a self-assessment of compliance with GATHER.

**Results**: Overall, WUENIC estimates are fully compliant with 17 of the GATHER criteria and partially compliant with one criterion—criterion 11, which is related to candidate model evaluation and final model selection.

**Conclusion**: The GATHER criteria provide a useful framework for documenting WUENIC’s compliance with contemporary reporting requirements. Given the role of vaccination coverage estimates in global monitoring and guiding disease control efforts, WHO and UNICEF strive to produce and publish robust estimates of vaccination coverage through a transparent process that emphasizes country involvement.

## Disclaimer

At the time this work was completed, M. Carolina Danovaro-Holliday and Marta Gacic-Dobo worked for the World Health Organization. The authors alone are responsible for the views expressed in this publication and they do not necessarily represent the decisions, policy or views of the World Health Organization. At the time this work was completed, Mamadou Diallo and Padraic Murphy worked for the United Nations Children’s Fund. The authors alone are responsible for the views expressed in this publication and they do not necessarily represent the decisions, policy or views of UNICEF.

## Introduction

In 2016, an expert working group, convened by the World Health Organization (WHO) to define and promote good practice in reporting of global health estimates, published the Guidelines for Accurate and Transparent Health Estimates Reporting (GATHER) ^1^. GATHER consists of a checklist of 18 essential items for reporting health estimates ( Table 1) and reflects a set of guidelines or best reporting practices for studies that calculate health estimates for multiple populations by defining minimum reporting requirements. Additional details are available on the GATHER website. The GATHER criteria aim to define and promote good practice in reporting health estimates. Compliance with GATHER is not an indicator of quality of the health estimates; rather, compliance implies that decision makers and researchers have the key pieces of information necessary for making informed judgements about the quality of the estimates. Statements of GATHER compliance have accompanied publications of global health estimates produced by the Joint United Nations Programme on HIV/AIDS (UNAIDS) ^2^ and the Malaria Atlas Project ^3^. In addition, compliance has also been noted in peer-reviewed papers ^4^.

**Table 1.** GATHER checklist criteria and WUENIC compliance status.

GATHER checklist criteria		WUENIC compliance rationale and status	
**1**	Define indicator(s), population(s) and time period(s) of estimates	Annual global, regional and national estimates and metadata (1980–2020) are available. *	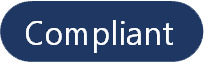
**2**	List the funding sources for the work	WUENIC is a core activity under respective agency mandates.	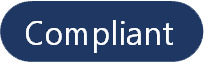
**3**	Describe how the data were identified and accessed	Input data are reported by country programmes and abstracted from survey reports.	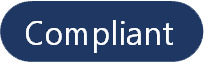
**4**	Specify inclusion and exclusion criteria	More detail required than can be provided here. See Ref 12.	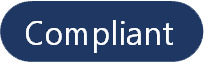
**5**	Provide information on all included data sources and their main characteristics	More detail required than can be provided here. See Ref 12.	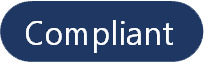
**6**	Identify and describe any input data that have potentially important biases.	More detail required than can be provided here. See Ref 12.	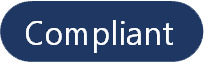
**7**	Describe and give sources for any other data inputs	Sources include the WHO/UNICEF Joint Reporting Form on Immunization † and consultation with regional and country-specific immunization experts.	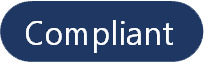
**8**	Provide all data inputs in a format from which data can be efficiently extracted	Data inputs are available in several electronic formats, including html and MS Excel. *	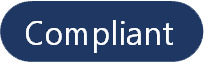
**9**	Provide a conceptual overview of the data analysis method	Country-by-country, vaccine-by-vaccine, year-by-year assessment of input data using a set of heuristic techniques expressed as rules and exceptions. No data borrowing from similar countries in the absence of data. No ad hoc adjustments. See Ref 12.	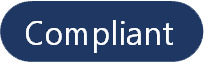
**10**	Provide a detailed description of all steps of the analysis	More detail required than can be provided here. See Ref 12.	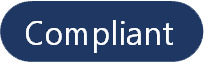
**11**	Describe how candidate models were evaluated and how the final model(s) were selected	More detail required than can be provided here. See Ref 12.	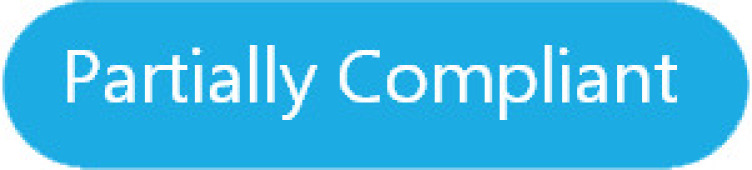
**12**	Provide the results of an evaluation of model performance and the results of any sensitivity analyses	Periodic, independent, external expert reviews have been conducted by QUIVER (2009, 2011), SAGE (2011) and IVIR-AC (2019). ‡	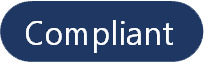
**13**	Describe methods for calculating uncertainty of the estimates	Approach is borrowed from artificial intelligence and uses an accumulation of endorsements or supporting information to produce a GoC that informs certainty for an estimated value.	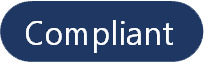
**14**	State how analytic or statistical code used to generate estimates can be accessed	Formal representation and Prolog code are available upon e-mail request from vpdata [at] who [dot] int.	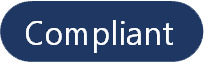
**15**	Provide published estimates in a file format from which data can be efficiently extracted	Published coverage estimates are publicly available. §	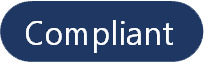
**16**	Report a quantitative measure of the uncertainty of the estimates	Published GoC values are publicly available. §	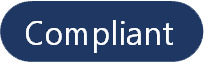
**17**	Interpret results in light of existing evidence. If updating a previous set of estimates, describe the reasons for changes in estimates	WUENIC is revised on an annual basis based on new and revised reported input data to reflect the most likely coverage given the available data.	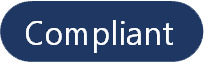
**18**	Discuss limitations of the estimates	More detail required than can be provided here. See Ref 12.	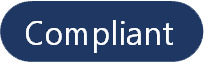

*Note:* GATHER: Guidelines for Accurate and Transparent Health Estimates Reporting; GoC: Grade of Confidence; IVIR-AC: Immunization and Vaccines related Implementation Research Advisory Committee; QUIVER: Quantitative Immunization and Vaccines Related Research, SAGE: Strategic Advisory Group of Experts on Immunization; WUENIC: WHO-UNICEF estimates of national immunization coverage (WUENIC). * Data may be accessed at: http://bit.ly/WUENICdata or https://data.unicef.org/topic/child-health/immunization. ^†^ Data may be accessed at: http://bit.ly/WHO-UNICEF-JRF. ^‡^ Data may be accessed at: WHO QUIVER, 2009: http://bit.ly/QUIVER2009; WHO QUIVER, 2011: http://bit.ly/QUIVER2011; WHO SAGE, 2011: http://bit.ly/SAGE2011; WHO IVIR-AC, 2019: http://bit.ly/IVIRAC2019. ^§^ Data may be accessed at: http://bit.ly/WUENIC-coverage.

Since 2001, the World Health Organization (WHO) and United Nations Children’s Fund (UNICEF) have jointly produced and published annual estimates of national vaccination coverage for all Member States of the World Health Assembly ^5^ and the State of Palestine. In addition to serving as a measure of an immunization programme’s ability to effectively deliver immunization services and protect against vaccine-preventable diseases, vaccination coverage for the recommended infant immunization series has been used to monitor the progress of global and regional immunization initiatives, including the Universal Childhood Immunization (UCI) by 1990 ^6^, the Decade of Vaccines and its Global Vaccine Action Plan ^7^ and the Immunization Agenda 2030 ^8^, endorsed by the World Health Assembly in August 2020. In addition, vaccination coverage is frequently used as an indicator of a health system’s ability to ensure access to primary care services and is currently included as one of many indicators in the Sustainable Development Goals ^9^.

During the 1980s and 1990s, WHO and UNICEF collected reports on vaccination coverage from Member States through separate, annual exercises. Country-specific coverage levels—based solely on the data reports from Member States—were published and used to produce regional and global estimates. In most instances, national coverage estimates were an aggregation of *1)* vaccinations given in the public sector and *2)* national-level population estimates or projections from the most recent census.

An internal review of global and regional coverage trends conducted in 1998 noted an apparent decline in global vaccination coverage based on national reports. Follow-up revealed that coverage decreases were not real but resulted from changes in the data sources being used by three countries, each with large annual birth cohorts. Specifically, coverage estimates from household surveys, rather than national administrative reports, were reported by India, Indonesia, and Bangladesh. Regional and global coverage appeared to decline because the survey reports consistently suggested meaningfully lower coverage as compared to administrative reports that had been previously used as the basis for reporting coverage.

Further examination of the consistency and reliability of national immunization coverage estimates reported to WHO from 1991 through 1996 ^10^ found that 25% of expected reports were missing; 20% of reported data points reflected year-to-year changes greater than 10 percentage points; roughly 15% of reports had a meaningful inconsistency between reported coverage for the third dose of diphtheria-tetanus-pertussis containing vaccine and the third dose of polio containing vaccine (vaccine doses that are generally recommended at the same age); and 17% of reports had a meaningful difference between coverage levels reported to WHO and coverage levels from other sources, namely population-based household surveys. The results were presented to WHO’s Strategic Advisory Group of Experts on Immunization (SAGE) ^11^, which recommended that WHO invest resources to improve the completeness, accuracy and precision of coverage estimates. Subsequently, methods were developed, data were identified and acquired, and an initial time series of WUENIC coverage estimates was produced in July 2001 for the period from 1980 through 1999. WUENIC has continued evolving since its inception 20 years ago.

## Methods

 Using the GATHER checklist as a reference, we conducted a desk review of all relevant documents and datasets maintained and produced by the WHO and UNICEF working group responsible for WUENIC to complete a self-assessment of compliance with the GATHER criteria. In the absence of structured guidance for conducting such an assessment of GATHER compliance, each author reviewed available material and made a self-assessment against three qualitative compliance categories (fully compliant, partially compliant, not compliant). Final determinations were exchanged within the group, disagreements discussed, and final revisions to methodological and process descriptions made and final compliance determined by group consensus.

## Results

This report summarizes WUENIC compliance with each of the 18 GATHER criteria. Detailed supporting descriptions are provided elsewhere ^12^. Overall, WUENIC estimates are fully compliant with 17 of the GATHER criteria and partially compliant with one criterion—criterion 11, which is related to candidate model evaluation and final model selection. GATHER is focused on estimates of health status and determinants rather than service coverage or health systems indicators ^13^ and inclined towards mathematical or statistical model-based approaches that synthesize data from multiple sources. Nonetheless, the GATHER criteria provide a useful framework for documenting WUENIC’s compliance with contemporary reporting requirements.

## WUENIC compliance with GATHER explained

The GATHER compliance checklist items and rationale for WUENIC compliance are summarized briefly below and are comprehensively described elsewhere ^12^.

*GATHER 1: Define indicators, populations (including age, sex, and geographic entities), and time period(s) for which estimates were made:* Vaccination coverage estimates are produced for 195 countries and territories. Annually, in July, estimates are published for the period from 1980 through the most recent calendar year (January through December). National vaccination coverage estimates reflect the crude percentage of infants vaccinated with a given vaccine dose combination (Table A1 in 12). In addition to global, population-weighted average coverage estimates, averages are produced for WHO regions, UNICEF regions, the World Bank income groupings and eligibility for support from Gavi, the Vaccine Alliance.

*GATHER 2: List funding sources for the work:* WHO and UNICEF are mandated to monitor and assess trends in the health and well-being of populations worldwide. The production of WUENIC estimates is funded in accordance with those mandates and through dues countries pay as members of the World Health Assembly and voluntary contributions from Member States and outside partners. Outside partners, including Gavi, the Vaccine Alliance and The Bill and Melinda Gates Foundation, have financially supported the production of WUENIC estimates.

*GATHER 3: Describe how the data were identified and accessed:* Immunization system performance reports, including vaccination coverage reports from national authorities, and survey data from published and grey literature are reviewed annually for 13 antigens (Table A1 in 12). Official coverage reflects a national authority’s assessment of coverage based on any combination of administrative coverage, survey-based estimates or other data sources or adjustments. Administrative coverage data are derived from routine administrative health service delivery reports. Vaccination coverage reports and other immunization system data are most often reported to WHO and UNICEF using the WHO and UNICEF Joint Reporting Form on Immunization (JRF). Survey-based coverage estimates are obtained from reports submitted by national authorities and from searches of national and survey agency websites (e.g., DHS; MICS).

Age-specific population estimates, which are utilized for producing global and regional averages and are an input to WUENIC’s measure of uncertainty, are obtained from the United Nations Population Division (UNPD) World Population Prospects site. Finally, WHO and UNICEF obtain additional otherwise unreported information, such as changes in immunization policies and insight into the functioning of the immunization system and quality of reported data, through consultation with regional and national immunization and monitoring experts.

*GATHER 4: Specify the inclusion and exclusion criteria:* Vaccination coverage data are included if they meet at least one inclusion criterion and do not meet any exclusion criterion and are excluded if they do not meet any inclusion criteria or meet at least one exclusion criteria. National administrative and official coverage must be reported to WHO and UNICEF in writing. Reported coverage levels >100% are excluded, and coverage levels that suggest large (>10 percentage point) year-to-year changes in vaccination coverage are excluded unless accompanied by an explanation. Dramatic increases or decreases in coverage are allowed for newly introduced vaccines.

Survey-based, vaccination coverage data must originate from finalized reports that include a sufficient description of the methods, including sampling methodology, in order to be included. Crude, but not valid, vaccination coverage estimates are considered. Survey results are excluded if they are not nationally representative; are derived from a survey of small size (i.e., <300 observations) or do not report sample size; or are not for single year cohorts (e.g., 12–23 months, 24–35 months).

*GATHER 5: Provide information on all included data sources and their main characteristics:* Countries report immunization system performance data annually using the WHO and UNICEF Joint Reporting Form on Immunization (JRF). These data include administrative and official vaccination coverage data and national vaccination schedule information. These data are complemented by population survey-based coverage data and by population data from the UNPD.

Official coverage reflects a national authority’s assessment of their most likely coverage based on any combination of administrative coverage, survey-based estimates or other data sources or adjustments. Administrative coverage data are derived from routine administrative health service delivery reports aggregated across service providers in the country. Survey coverage reflects an assessment of coverage during a specified time period from a defined sample population that is independent of the biases in numerator and denominator data described below. National vaccination schedules provide standardized information regarding the vaccines and number of doses of vaccine that a population should receive and the recommended ages, minimum ages and intervals between doses. Vaccination schedules also provide information on whether a vaccine is recommended for an entire population or targeted to specific risk groups. In addition, population data from UNPD provide a consistent reference population time series for computing global and regional average coverage levels.

*GATHER 6: Identify and describe any categories of input data that have potentially important biases:* Both the reported number of administered vaccine doses and the reported target population used to compute administrative coverage may be biased, which may lead to biased coverage estimates. Biases affecting numerator data include, but are not limited to, incomplete or untimely reporting and data recording or reporting errors. Biases affecting denominator data include, but are not limited to, population estimates based on dated or poorly implemented census estimates or poorly implemented census projections. The methods and data sources used to produce official estimates are not always described or available, and methods may change over time or without documentation or notice. When a country bases its official coverage on administrative coverage, biases in the administrative coverage carry over to official coverage. Survey-based coverage estimates are subject to sampling and non-sampling error.

*GATHER 7: Describe and give sources for any other data inputs:* WHO and UNICEF use data on new vaccine introductions, vaccine stockouts, the occurrence of mass vaccination events and vaccine preventable disease surveillance. Data for each of these are obtained from country reports to WHO and UNICEF using the JRF. New vaccine introduction data, alongside reported coverage data, help inform when WUENIC should begin calculating estimates. Data are reported on the occurrence and duration of vaccination stockouts at national and subnational levels and may help explain year-to-year changes in coverage levels. The occurrence of mass vaccination events is useful contextual information for discerning whether coverage levels might reflect campaign rather than routine vaccination delivery.

*GATHER 8: Provide all data inputs in a format from which data can be efficiently extracted:* All input data are publicly available on the WHO website in MS Excel format, including “4.1 Official country reported coverage estimates time series”, “4.2 Administrative data time series”, and “4.7 Coverage Survey Data” with abstracted results from population-based survey reports.

*GATHER 9: Provide a conceptual overview of the data analysis method:* WHO and UNICEF distinguish between when data reported by national authorities accurately reflects immunization system performance and when the data are likely compromised or misleading. Unless challenged, the nationally reported estimate constitutes the WUENIC. If reported data are supported by independent survey results, then WUENIC will reflect the reported data. When reported data are challenged by survey results, WUENIC may diverge from the reported data.

WUENIC are based on a country-by-country, vaccine-by-vaccine, year-by-year assessment of available data. The estimates are not the product of a formal modelling exercise; no statistical or mathematical models are used (with one exception related to estimates for the first dose of diphtheria-tetanus-pertussis containing vaccine to ensure that estimated coverage for the third dose does not exceed that for the first dose). Data from different sources are not averaged to arrive at a final estimate, and the approach does not borrow information from data rich countries to fill in gaps among data poor countries. A detailed description is available in Burton *et al.* ^14^ and Burton *et al.* ^15^.

*GATHER 10: Provide a detailed description of all steps in the analysis:* Briefly, the aim of the WUENIC process is to produce a time series of annual vaccination coverage estimates for selected vaccines given data reported by national authorities and given available nationally representative survey data and any other available information. The estimation process is comprised of four general steps: *1)* accepting, adjusting or ignoring available reported and survey coverage data; *2)* generating coverage estimates for years when both reported and survey data are available (so-called “anchor” years); *3)* generating coverage estimates for non-anchor years in which there is no survey data; and *4)* examining estimates for year-to-year and vaccine-to-vaccine consistency and reconciling any discrepancies. Stepwise visual descriptions of the WUENIC estimation process are provided in Brown and colleagues ^12^.

*GATHER 11: Describe how candidate models were evaluated and how the final model(s) were selected:* Since the initial release in 2000, the WUENIC estimation process and methods have been periodically reviewed by independent, external expert panels, including the WHO Quantitative Immunization and Vaccines Related Research (QUIVER) Advisory Committee (2009 ^16^, 2011 ^17^), the WHO Strategic Advisory Group of Experts on Immunization (SAGE; 2011 ^18^), and the WHO Immunization and Vaccines Related Implementation Research Advisory Committee (2019 ^19^). Reviews have supported the approach. WHO is working with the Institute for Health Metrics and Evaluation (IHME) to compare and contrast coverage estimates derived from a space-time Gaussian process regression model with WUENIC estimates.

*GATHER 12: Provide results of an evaluation of model performance, if done, as well as the results of any relevant sensitivity analysis:* Independent, external expert reviews have supported the WUENIC approach. Results are available for QUIVER 2009 ^16^; QUIVER 2011 ^17^; SAGE 2011 ^18^; IVIR-AC 2019 ^19^.

*GATHER 13: Describe methods of calculating uncertainty of the estimates. State which sources of uncertainty were, and were not, accounted for in the uncertainty analysis:* WUENIC introduced a Grade of Confidence (GoC) with the 2011 revision (completed July 2012) to communicate uncertainty ^20^. The GoC reflects the accumulation of endorsements, or sources of supporting information, that influence certainty about an estimated value. GoC values range from one (low confidence in an estimate) to three (high confidence in an estimate). The GoC does not reflect the quality of the underlying input data. All WUENIC estimates carry a risk of being incorrect given potential deficiencies in the input data.

*GATHER 14: State how analytic or statistical source code used to generate estimates can be accessed:* A computational logic-based representation of rules, data and decisions for WUENIC is provided in Burton *et al.* ^15^.

*GATHER 15: Provide published estimates in a file format from which data can be efficiently extracted:* Complete time series of country-specific, regional and global coverage estimates are available in electronic file formats (HTML, MS Excel, and PDF) available on the WHO website ^21^.

*GATHER 16: Report a quantitative measure of the uncertainty of the estimates:* Because there is no underlying probability distribution for the WUENIC estimates, classic quantitative measures of uncertainty, such as confidence intervals, are not used. The GoC is a qualitative measure of uncertainty (see checklist item 13), which is published alongside the WUENIC time series from 1997 through the most recent release and available on the WHO website ^21^.

*GATHER 17: Interpret results in light of existing evidence:* Annual estimate production involves a revision of the time series to reflect new data as well as updated data for prior years. New or updated data may include reports by national authorities, estimates from surveys, contextual information, methodological updates, and updated population estimates from UNPD. As such, coverage levels for the current revision are not comparable to those from previous revisions.

*GATHER 18: Discuss limitations of the estimates:* Estimates are limited by the quality of the underlying empirical input data. Inaccuracies may exist within the reported numerator and denominator of administrative or official coverage estimates. Vaccination coverage estimates from population-based household surveys may be limited by the quality of the planning and implementation of the field work and the availability of documented evidence of vaccination history in home-based or facility-based records. Additionally, coverage indicators from survey reports do not always conform to standard definitions. WUENIC is also constrained by the underlying rules and heuristics utilized in the estimation approach.

## Conclusions

Boerma and colleagues ^22^ described the role of health estimates such as WUENIC in monitoring progress towards global and regional goals and targets and guiding resource allocation while emphasizing the importance of transparency in the production of these health statistics. This report summarizes compliance of the WHO and UNICEF estimates of national immunization coverage with each of the 18 GATHER criteria for population health estimates. Although the GATHER guidelines were not originally designed for health service delivery indicators, such as vaccination coverage, they provide a useful framework for documenting WUENIC’s compliance with contemporary reporting requirements. Given the role of vaccination coverage estimates in monitoring immunization system performance, guiding disease control efforts, and informing assessments of high risk areas that might require additional resources, WHO and UNICEF strive to produce and publish robust estimates of vaccination coverage through a transparent process that emphasizes country involvement. We acknowledge the strategic advantages of transparency and provide this summary of WUENIC compliance with GATHER in commitment to promoting openness so that health estimates such as WUENIC vaccination coverage estimates are translated in a meaningful way to promote global vaccination goals.

## Data availability

### Underlying data

Zenodo. Description of WHO and UNICEF estimates of national immunization coverage compliance with GATHER criteria, https://doi.org/10.5281/zenodo.4730579 ^12^.

This project contains the following data:

- a detailed description of compliance of the WHO and UNICEF estimates with the Guidelines for Accurate and Transparent Health Estimates Reporting (GATHER) criteria.

Data are available under the terms of the Creative Commons Zero "No rights reserved" data waiver (CC0 1.0 Public domain dedication).
