# Supplementary material for: Compliance of WHO and UNICEF estimates of national immunization coverage (WUENIC) with Guidelines for Accurate and Transparent Health Estimates Reporting (GATHER) criteria
Source: Gates Open Res. 2021 May 10;5:77. [Version 1] doi: 10.12688/gatesopenres.13258.1 (PMC9021403; doi:10.12688/gatesopenres.13258.1)
Supplement: Peer review history

# Peer review: Reviewer response for version 1

**DOI:** 10.21956/gatesopenres.14493.r30909

**Published:** 2021-08-03

**Authors:** Amalie Dyda

**Affiliations:** 1 School of Public Health, Faculty of Medicine, The University of Queensland, Herston, Qld, Australia

This is an extremely well written paper and an important addition to the literature. It outlines a comparison of the WHO and UNICEF estimates of national immunization coverage (WUENIC) against the Guidelines for Accurate and Transparent Health Estimates Reporting (GATHER) to assess agreement between the two. This is important given the importance of, but many challenges of, reporting global annual national vaccination coverage.

 Of serious note is the possible error/bias in how each coverage estimate is collected. I think the reason for the importance of using GATHER has been highlighted in the introduction ("Compliance with GATHER is not an indicator of quality of the health estimates; rather, compliance implies that decision makers and researchers have the key pieces of information necessary for making informed judgements about the quality of the estimates."), and bias addressed well in Gather 6.

 I have provided a few specific comments for consideration below:

**Methods:**

 "In the absence of structured guidance for conducting such an assessment of GATHER compliance, each author reviewed available material and made a self-assessment against three qualitative compliance categories (fully compliant, partially compliant, not compliant)."

 I believe this authorship group has the required experience and knowledge to provide this expert assessment. I think this would be strengthened by either adding a sentence to highlight this, or providing some more detailed information about what authors took into consideration in their reviews.

**Results:**

 "GATHER 10: Provide a detailed description of all steps in the analysis:"

 This description of the steps was quite brief, but given the context of what is needed in this comparison it is sufficient, especially given more detailed methods are referenced.

**Conclusion:**

 "Boerma and colleagues described the role of health estimates such as WUENIC in monitoring progress towards global and regional goals and targets and guiding resource allocation while emphasizing the importance of transparency in the production of these health statistics."

 Perhaps better placed in the introduction.

Is the work clearly and accurately presented and does it cite the current literature?

Yes

If applicable, is the statistical analysis and its interpretation appropriate?

Not applicable

Are all the source data underlying the results available to ensure full reproducibility?

No source data required

Is the study design appropriate and is the work technically sound?

Yes

Are the conclusions drawn adequately supported by the results?

Yes

Are sufficient details of methods and analysis provided to allow replication by others?

Partly

Reviewer Expertise:

infectious disease epidemiology, vaccination coverage, public health informatics

I confirm that I have read this submission and believe that I have an appropriate level of expertise to confirm that it is of an acceptable scientific standard.

# Peer review: Reviewer response for version 1

**DOI:** 10.21956/gatesopenres.14493.r30903

**Published:** 2021-07-27

**Authors:** Fuqiang Cui

**Affiliations:** 1 Department of Laboratorial Science, School of Public Health, Peking University, Beijing, China

The study validated the compliance of WUENIC with the 18 GATHER criteria, it presents clearly and accurately results with reasonable clarifications. Among each GATHER criterion, the explanation is fully supported by the data and evidence.

 While I agreed with the study design and results overall, I still hope for some clarifications in methods:

1. First, the authors conducted a desk review of all relevant documents and datasets, I wonder if readers wanted to know what the relevant documents and datasets are?
2. Second, among 18 GATHER criteria, the authors defined the category into fully compliant, partially compliant, not compliant - for me, this is less clear, does this mean 100%, 1-99%, and 0% compliant? Please clarify.
3. Third, I understood there is some incompleteness in terms of the reported data, the global compliance does not mean it is applicable at regional or national level, this has to be discussed. Of course, these are some tiny suggestions, and does not influence the conclusions drawn adequately supported by the results.

I believe the paper has academic merit, please take actions if it makes sense or ignore it if you could fully clarify. In any case, I would approve this study to be indexed soon.

Is the work clearly and accurately presented and does it cite the current literature?

Yes

If applicable, is the statistical analysis and its interpretation appropriate?

Yes

Are all the source data underlying the results available to ensure full reproducibility?

Partly

Is the study design appropriate and is the work technically sound?

Yes

Are the conclusions drawn adequately supported by the results?

Yes

Are sufficient details of methods and analysis provided to allow replication by others?

Yes

Reviewer Expertise:

Vaccine, epidemiology, infectious diseases

I confirm that I have read this submission and believe that I have an appropriate level of expertise to confirm that it is of an acceptable scientific standard.
